# Pharmacological augmentation of Cognitive Remediation for Schizophrenia: a systematic review

**DOI:** 10.1192/j.eurpsy.2026.10544

**Published:** 2026-07-31

**Authors:** A. Ceraso, R. Alberti, R. Virgillito, M. Fiore, A. Rajkovic, F. Coronelli, E. De Leo, S. De Bellis, L. Pascariello, U. M. Battelino, G. Nibbio, G. Deste, S. Barlati, A. Vita

**Affiliations:** 1 Department of Mental health and addiction services, Asst Spedali Civili Brescia; 2 Department of Clinical and Experimental Sciences, University of Brescia, Brescia, Italy

## Abstract

**Introduction:**

Cognitive impairments are observed in 75-80% of people with Schizophrenia, with a significant impact on everyday functioning, quality of life, treatment adherence, costs of care and rehabilitation outcomes.

**Objectives:**

The primary objective is to examine the efficacy of the combination of cognitive rehabilitation and pharmacological treatments with potential pro-cognitive action compared to stand-alone interventions. The next aims are to analyze the applicability context, the limitations of available scientific evidence and to determine future perspectives.

**Methods:**

This study follows the guidelines of PRISMA Statement and of Cochrane Collaboration. The systematic research was conducted on electronic databases: PubMed, PsychINFO, Scopus, Cochrane CENTRAL. English, peer-reviewed papers were selected. A manual search was performed on Google Scholar. At least two revisors examined independently all papers. Methodological quality of included studies was evaluated using Clinical Trial Assessment Measure (CTAM). Inclusion criteria: randomized controlled trials (RCT) with baseline and at post-treatment outcome assessments, regardless of setting or duration; patients with diagnosis of Schizophrenia spectrum disorders, clinically stable and stabilized on antipsychotic therapy; combination of a cognitive rehabilitation intervention with a molecule with pro-cognitive function, tested against control groups treated with cognitive rehabilitation or with pro-cognitive molecules administered as stand-alone treatments; evaluation of efficacy on cognitive functioning, on psychosocial functioning and clinical symptoms, with validated assessment measures.

**Results:**

3126 studies were identified through electronic databases, 2073 excluding duplicates. 143 articles were identified with other resources. Totally 2216 papers were screened, 98 were examined and 25 papers related to 20 studies were included. Molecules investigated: atypical antipsychotics, d-serine, d-cicloserine, oxiytocin, modafinil, nicotine.

**Conclusions:**

This review shows that available data are limited to specific settings of care and this might affect the results. The efficacy of combined cognitive rehabilitation and specific molecules on clinically stable individuals with schizophrenia is largely inconclusive. However, this issue is scientifically and clinically a topic of interest, warranting further research.

**Disclosure of Interest:**

A. Ceraso: None Declared, R. Alberti: None Declared, R. Virgillito: None Declared, M. Fiore: None Declared, A. Rajkovic: None Declared, F. Coronelli: None Declared, E. De Leo: None Declared, S. De Bellis: None Declared, L. Pascariello: None Declared, U. Battelino: None Declared, G. Nibbio: None Declared, G. Deste Consultant of: -, S. Barlati Consultant of: -, A. Vita Consultant of: -.

